# The epidermal growth factor receptor regulates cofilin activity and promotes transmissible gastroenteritis virus entry into intestinal epithelial cells

**DOI:** 10.18632/oncotarget.7723

**Published:** 2016-02-25

**Authors:** Weiwei Hu, Liqi Zhu, Xing Yang, Jian Lin, Qian Yang

**Affiliations:** ^1^ Veterinary College, Nanjing Agricultural University, Nanjing, Jiangsu, PR China; ^2^ Life Science College, Nanjing Agricultural University, Nanjing, Jiangsu, PR China

**Keywords:** TGEV, F-actin, cofilin, EGFR, Immunology and Microbiology Section, Immune response, Immunity

## Abstract

Transmissible gastroenteritis virus (TGEV), a coronavirus, causes severe diarrhea and high mortality in newborn piglets. The porcine intestinal epithelium is the target of TGEV infection, but the mechanisms that TGEV disrupts the actin cytoskeleton and invades the host epithelium remain largely unknown. We not only found that TGEV infection stimulates F-actin to gather at the cell membrane but the disruption of F-actin inhibits TGEV entry as well. Cofilin is involved in F-actin reorganization and TGEV entry. The TGEV spike protein is capable of binding with EGFR, activating the downstream phosphoinositide-3 kinase (PI3K), then causing the phosphorylation of cofilin and F-actin polymerization via Rac1/Cdc42 GTPases. Inhibition of EGFR and PI3K decreases the entry of TGEV. EGFR is also the upstream activator of mitogen-activated protein kinase (MAPK) signaling pathways that is involved in F-actin reorganization. Additionally, lipid rafts act as signal platforms for the EGFR-associated signaling cascade and correlate with the adhesion of TGEV. In conlusion, these results provide valuable data of the mechanisms which are responsible for the TGEV pathogenesis and may lead to the development of new methods about controlling TGEV.

## INTRODUCTION

Porcine transmissible gastroenteritis virus (TGEV) is an enveloped enteropathogenic coronavirus (CoV) with a large positive-sense single-stranded RNA genome about 28.5 kb in length. Similar to other CoVs, TGEV has a diameter ranging from 100 to 150 nm, including the surface projections. TGEV infects newborn piglets with mortality rates reaching 100%, resulting in significant losses in the swine industry. The CoV spike protein binds to a cellular receptor and then mediates membrane fusion at the plasma membrane or by endosomal uptake. TGEV, Human coronavirus (HCoV-229E), serotype 2 Feline Coronavirus (FCoV), and Canine Coronavirus (CCoV) all use the aminopeptidase N (APN) protein as the receptor [1]. TGEV infects epithelial cells through the small intestine and the respiratory tract. The virus enters epithelial cells from the apical or basolateral side, and is released from the apical plasma membrane into the gut lumen, where it propagates efficiently by cell-to-cell spreading [2]. Porcine intestinal columnar epithelial cells (IPEC-J2) offer a practical model for studying porcine enteric pathogens [3]. However, the precise molecular mechanisms responsible for TGEV entry are largely unknown, and limited informations are available on the cell signaling pathways involved in coronavirus entry *via* the actin cytoskeleton.

Located beneath the plasma membrane, cortical actin is composed of a loosely organized network of actin cytoskeleton that is highly dynamic and is involved in many cellular processes. Many pathogens facilitate cell entry and/or trafficking by stimulating actin remodeling [4, 5]. Cofilin plays an important role in actin polymerization and depolymerization [6]. LIM-kinases (LIMKs) inhibit the activity of cofilin by phosphorylating the serine residue at position 3 (Ser-3). LIMKs are activated by Rho-associated kinase (ROCK), p21- activated protein kinases (PAKs), which are downstream kinases of the Rho family GTPases, RhoA, Rac1, and Cdc42 [7]. Rho GTPases regulate actin polymerization, induce plasma membrane protrusion and control vesicle trafficking [8]. The phosphoinositide-3 kinase (PI3K) pathway is activated by a variety of extracellular stimuli and regulates a wide range of cellular processes, including cell cycle progression, cell growth, cell motility, cell adhesion and vesicular trafficking [9, 10]. The serine/threonine kinase (Akt) is a central node in cell signaling downstream of growth factors, cytokines, and other cellular stimuli [11]. Receptor tyrosine kinases (RTKs) play an important role in transforming extracellular intracellular signals and activate PI3K as well as extracellular signal regulated kinase (ERK)1/2 [12]. The epidermal growth factor receptor (EGFR) belongs to the RTK family, and is activated by a family of growth factors including epidermal growth factor (EGF), transforming growth factor-α (TGF-α), and the neuregulins. It also interacts with three homologous transmembrane proteins ErbB2, ErbB3 and ErbB4 [13, 14]. The binding of EGF to its cell surface receptor activates the receptor's intrinsic tyrosine kinase and phosphorylates the tyrosine at its C-terminus. Phosphorylated EGFR is essential for the activation of Ras GTPase and ERK [15]. EGFR can be activated by many viruses, including influenza A, hepatitis C (HCV), Herpes simplex type 1(HSV-1), and human cytomegalovirus (HCMV) [16-19].

In this study, we found that TGEV caused F-actin rearrangement and membrane ruffling early in infection. The phosphorylation of the EGFR was also detected early in infection. We found that TGEV acted *via* the EGFR-PI3K-Rac1/Cdc42-PAK-LIMK signaling pathway to regulate the activity of cofilin and F-actin arrangement early in infection, and also demonstrated that EGFR was a promoter for TGEV entry.

## RESULTS

### TGEV induces cell plasma membrane extensions and biphasic regulation of cofilin activity

**Figure 1:**
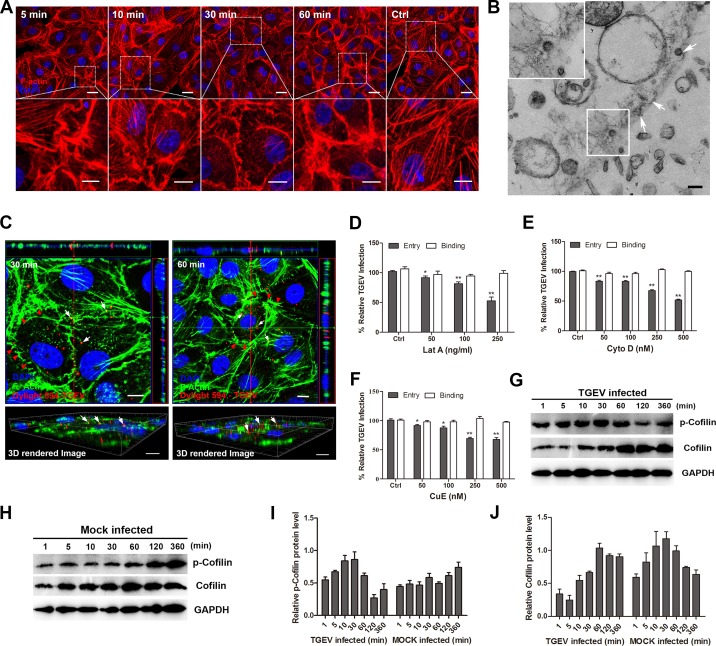
Actin cytoskeleton dynamics are crucial for TGEV entry **A.** IPEC-J2 cells were incubated with TGEV (MOI = 2) at 4°C for 1 h, shifted to 37°C, and then fixed at the indicated time points. F-actin was stained with phalloidin-TRITC (Red) and observed by confocal microscopy. Scale bar = 20 μm. **B.** Electron microscopic analysis of ultrathin sections of IPEC-J2 cells infected with TGEV (MOI = 10), the white arrows indicated the podosome and lamellipodium. Scale bar = 150 μm. **C.** F-actin surround with TGEV particles. TGEV particles were labeled with fluorescent probe Dylight 594, IPEC-J2 cells were incubated with DyLight 594 labeled TGEV at 4°C for 1 h, then shifted to 37°C, fixed at 30 mpi and 60 mpi, F-actin stained with phalloidin (Green). Images were captured with a Zeiss LSM710 confocal laser-scanning microscopy system and rendered three-dimensional (3D) images. Scale bar = 10 μm. **D.** to **F.** Concentration-dependent inhibition of TGEV (MOI = 2) entry by cytoskeleton inhibitors. **G.** and **H.** Cells were incubated with TGEV (MOI = 2) at 4°C for 1 h, unbound virus removed, and cells were then incubated at 37°C. Levels of p-cofilin, cofilin and p-LIMK were measured by Western blotting using either mAb specific for p-cofilin, or pAb for p-LIMK, cofilin. **I.** and **J.** The amount of p-cofilin and cofilin were quantified. Statistical significance was assessed by Student's *t*-test. Differences were considered significant at (*) 0.01 < *p* < 0.05, (**) *p* < 0.01. All experiments were performed separately three times.

Actin cytoskeleton assembly/disassembly dynamics are critical for many endocytic pathways [20]. In order to explore potential interactions between TGEV and F-actin, we stained cells shortly after infection with phalloidin-TRITC and examined them using confocal microscopy (Figure 1A). At 5 min post-infection (mpi), F-actin filaments were observed close to the cell plasma membrane, and accumulated in this region as the experiment progressed. At 30 mpi, actin stress fibers had became noticeably less abundant in the cytoplasm. At 60 mpi, almost all F-actin was at the cell membrane. Transmission electron microscopy (TEM) confirmed that F-actin gathered underneath the plasma membrane, the podosome and lamellipodium were also observed in the cell membrane (Indicated by the white arrows) (Figure 1B).

The relationship between TGEV particles and F-actin early in infection were further examined by confocal fluorescence microscopy, with infected cells stained using phalloidin-TRITC, TGEV particles were labeled with fluorescent probe DyLight 594 (Figure 1C). At 30 mpi, F-actin was organized to produce a range of cell surface protrusions (red arrows), TGEV particles had internalized into the cell membrane and begun to enter the cytoplasm. At 60 mpi, TGEV had entered the cytoplasm, we found that F-actin surrounded the TGEV particles in spatial, part of the virus co-localized with the F-actin (yellow) (white arrows). These phenomena were also shown in 3D rendered images. Together, these results suggest that the process of TGEV infection causes F-actin accumulation around the cell membrane, possibly promoting viral binding, penetration and intracellular trafficking.

To test these hypothesis, IPEC-J2 cells were pretreated with three cytoskeleton inhibitors: Latrunculin A (Lat A), Cytochalasin D (Cyto D), and Cucurbitacin E (Cu E) at 37°C for 1h. The effect of these inhibitors on cell viability was shown in Figure S1. All three inhibitors reduced TGEV entry in a dose dependent manner compared with mock control cells (Figure 1D, 1E, 1F). These results provide evidence that F-actin is involved in the TGEV entry process.

To investigate the relationship between cofilin and TGEV infection, we measured the levels of p-cofilin and cofilin in infected and uninfected cells by Western blot (Figure 1G, 1H, 1I, 1J). In TGEV-infected cells, the level of p-cofilin increased from 1 mpi to 30 mpi and decreased at 60 mpi, but the level of cofilin was low from 1 mpi to 30 mpi and increased at 60 mpi. In uninfected cells, the level of p-cofilin was low from 1mpi to 60 mpi and increased at 120 mpi, but the level of cofilin maintained a high expression level from 5mpi to 60 mpi. We hypothesize that the process by which TGEV causes F-actin filaments to gather around the cell membrane and the formation of cell surface protrusions require the participation of p-cofilin.

### Cofilin regulates the actin cytoskeleton early in TGEV infection

**Figure 2:**
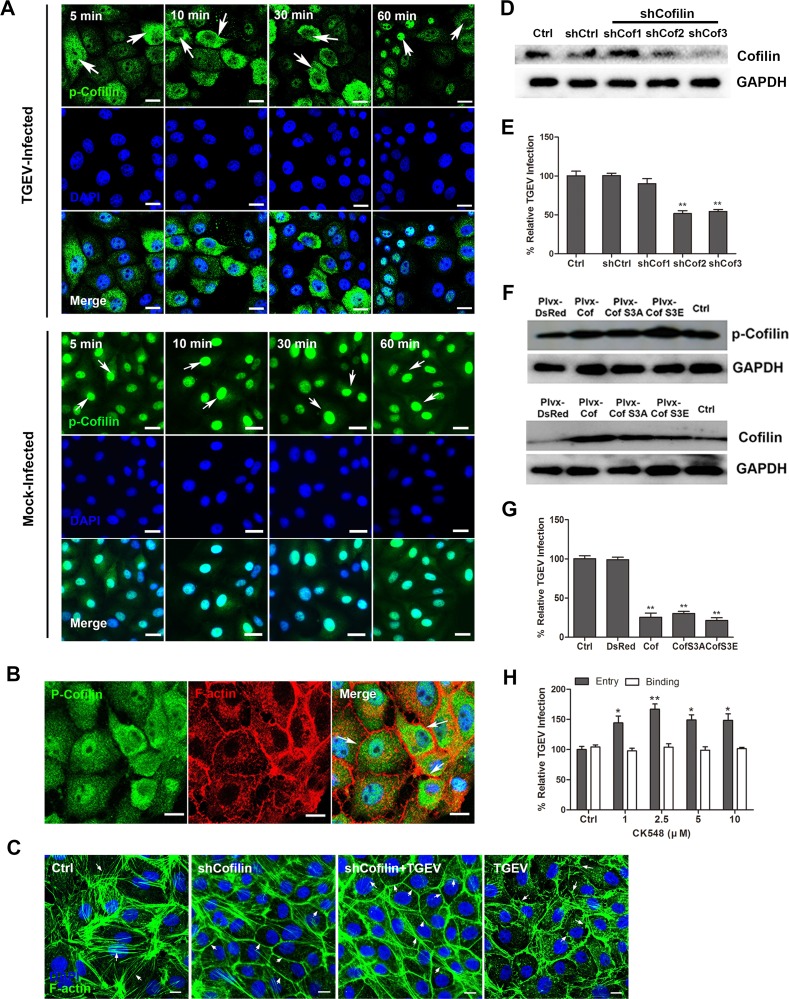
Role of cofilin in TGEV-mediated rearrangement of actin filaments **A.** Levels of p-cofilin increase early in TGEV infection. Cells were fixed at the indicated time points, stained with anti-p-cofilin antibody, and observed by confocal microscopy. Scale bar = 10μm. **B.** The distribution of p-cofilin and F-actin early in TGEV infection. At 30 mpi, cells were fixed and stained with anti-p-cofilin antibody, followed by Dylight 488-conjugated goat anti-rabbit IgG. F-actin was stained with phalloidin-TRITC (Red). Scale bar = 10μm. **C.** Cofilin shRNA block the destruction of actin cytoskeleton caused by TGEV infection. Cells were incubated with cofilin shRNA for 48h, cells were infected with TGEV for 1 h, F-actin was stained with phalloidin-TRITC (Green), Scale bar = 10μm. **D.** Cofilin shRNA verification. Cells were transfected with three cofilin shRNAs. After 48 h incubation, the expression of cofilin was verified by Western blotting. **E.** Cofilin shRNAs block TGEV entry. Cells were transfected with lentiviral vectors expressing cofilin shRNAs. After 48 h incubation, cells were infected with TGEV for 1 h and entry of TGEV was evaluated by real-time PCR. **F.** Verification of expression of WT cofilin and mutant cofilins S3A and S3E. Cells were treated with lentiviral particles expressing WT cofilin or mutant cofilins S3A and S3E. After 48 h incubation, expression of p-cofilin and cofilin were verified by Western blotting. **G.** The WT cofilin and mutant cofilins S3A and S3E block TGEV entry. Cells were treated with lentiviral particles expressing WT cofilin or mutant cofilins S3A and S3E. After 48 h incubation, cells were infected with TGEV for 1 h and entry of TGEV was evaluated by real-time PCR. **H.** ARP2/3 inhibitor CK548 promotes TGEV entry. IPEC-J2 cells were pretreated with CK548 at different concentrations at 37°C for 1 h, for details concerning binding and entry assays, see materials and methods. Statistical significance was assessed by Student's *t*-test. Differences were considered significant at (*) 0.01 < *p* < 0.05, (**) *p* < 0.01. Scale bar = 20 μm. TGEV at a multiplicity of infection of 2 (MOI = 2).

To examine the role of cofilin more closely, we conducted series of experiments in which cofilin distribution and function were assessed in uninfected and TGEV-infected cells. Most p-cofilin was found in the nucleus of uninfected cells (Figure 2A). In contrast, early in TGEV infection, p-cofilin redistributed to the cytoplasm and was less abundant in the nucleus. However, at 60 mpi, p-cofilin appeared to shift from the cytoplasm back to the nucleus (Figure 2A). When the distribution of p-cofilin and F-actin were observed early in infection, we found most p-cofilin did not colocalize with F-actin, and multiple protrusions were noted around the cell membrance (Figure 2B). We knocked cofilin down in normal IPEC-J2 cells, and found that the actin stress fibers were also in the cytoplasm. Usually underneath the cell membrane are the loosely organized network of F-actin filaments that are termed cortical actin [4], but the cortical actin underneath the cell membrane gathered together, the cell surface was smooth and couldn't form protrusions. In cofilin knockdown cells, early in TGEV-infection, some actin stress fibers were also found in the cytoplasm, the protrusions in the cell membrance reduced significantly. The transient increased in p-cofilin level in the cytoplasm from 1 mpi to 30 mpi correlated with the F-actin filaments around the cell membrane and the formation of multiple protrusions. All of these results demonstrate that cofilin involve in the regulation of F-actin early in TGEV infection.

To confirm the role of cofilin in TGEV infection, we used molecular methods to modify the levels of cofilin and p-cofilin prior to infection by TGEV. Cells were transfected with cofilin targeting shRNAs, the cofilin expression level was decreased significantly (Figure 2C), and TGEV entry level was decreased (Figure 2D). Cells were also transfected with vectors expressing WT cofilin, constitutively non-phosphorylated mutant cofilin (activated, S3A), or constitutively-phosphorylated mutant cofilin (inactivated, S3E) [21, 22]. The expression of WT or constitutively inactivated cofilin S3E significantly increased the expression of p-cofilin (Figure 2E). All three constructs inhibited TGEV entry (Figure 2F). Examination using confocal fluorescence microscopy showed that in uninfected cells, the F-actin partially depolymerized in WT cofilin-expressing cells and in constitutively activated cofilinS3A-expressing cells, while the F-actin in constitutively inactivated cofilinS3E-expressing cells, polymerized around the cell membrane (Figure S2).

We also explored the role of actin-related protein 2/3 (ARP2/3) in TGEV entry using its specific inhibitor CK548. Cells were treated with different concentrations of CK548 at 37°C for 1h prior to TGEV infection. CK548 did not inhibit virus binding, but promoted TGEV entry (Figure 2G).

### Rac1 and Cdc42 GTPase are involved in cofilin phosphorylation early in TGEV infection

**Figure 3:**
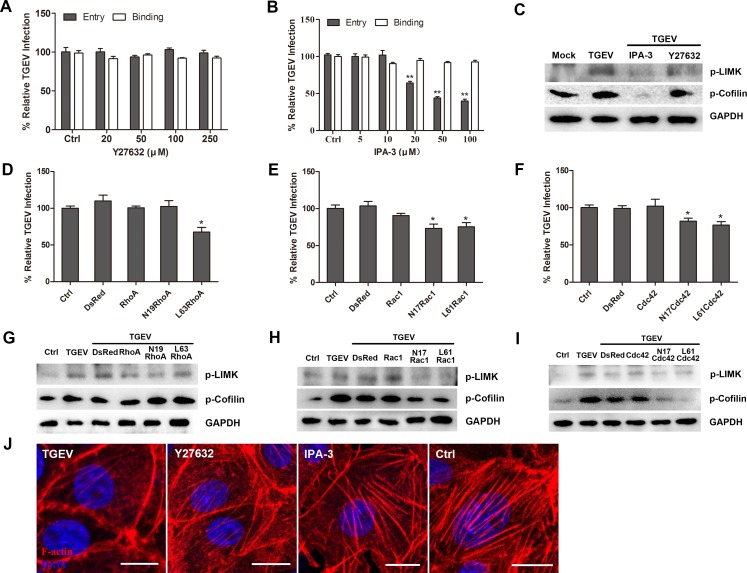
Rac1GTPase and Cdc42GTPase are involved in the early cofilin phosphorylation **A.** ROCK inhibitor Y27632 has no effect on the entry of TGEV. IPEC-J2 cells were pretreated with Y27032 at different concentrations at 37°C for 1 h. TGEV binding and entry assays are described in materials and methods. **B.** PAKs inhibitor IPA-3 does inhibit TGEV entry. IPEC-J2 cells were pretreated with IPA-3 at different concentrations at 37°C for 1 h. **C.** PAKs are involved in the phosphorylation of cofilin. Cells were pretreated with Rock inhibitor Y27632 (100μM) and PAKs inhibitor IPA-3 (50μM) at 37°C for 1 h. 30 min after TGEV infection, cell lysates were analyzed for phosphorylation of LIMK and Cofilin. (D to F) RHO-family-GTPases are involved in the entry of TGEV. Cells were treated with lentiviral particles expressing WT, constitutively-activated, and constitutively-inactivated RHO GTPases. After 48 h incubation, cells were infected with TGEV for 1 h. Entry of TGEV was evaluated by real-time PCR. (G to I) Rac1 and Cdc42 are involved in the regulation of cofilin. Cells were treated with lentiviral particles expressing WT, constitutively-activated, and constitutively-inactivated RHO GTPases. After 48 h incubation, cells were infected with TGEV for 30 min and cell lysates were analyzed for the phosphorylation of LIMK and Cofilin. **J.** IPA-3 protects the actin cytoskeleton. Cells were treated with Y27632 (100 μM), IPA-3 (50 μM) at 37°C for 1 h, then infected with TGEV. 1 h after TGEV infection, cells were fixed and stained with phalloidin-TRITC and observed by confocal fluorescence microscopy. TGEV at a multiplicity of infection of 2 (MOI = 2).

We pretreated cells with several RHO-family-GTPases inhibitors prior to TGEV infection to determine whether some were the upstream regulators of cofilin. ROCK inhibitor Y27632 did not inhibit TGEV binding as well as TGEV entry (Figure 3A). PAKs inhibitor IPA-3 also had no effect on TGEV binding, but decreased TGEV entry in a dose dependent manner (Figure 3B). IPA-3 inhibited LIMK activation and cofilin phosphorylation, while ROCK inhibitor Y27632 had no inhibitory effect (Figure 3C). Using confocal fluorescence microscopy, we also observed that IPA-3 appeared to inhibit the destruction of F-actin by TGEV infection, but LY294002 couldn't inhibit the destruction of F-actin by TGEV infection (Figure 3J). The effect of these inhibitors on cell viability was shown in Figure S1.

In order to study the role of RHO-family-GTPases early in the entry process of TGEV, cells were transfected with lentivirus constructs that expressed wild type, constitutively-activated, or constitutively-inactivated RHO-family-GTPases. The expression of constitutively activated L63RhoA, L61Rac1, and L61Cdc42, as well as constitutively inactivated N17Rac1 and N17Cdc42, decreased TGEV entry (Figure 3D, 3E, 3F). Additionally, some RHO-family-GTPases affected p-cofilin level early in TGEV infection, the level of p-cofilin was significantly inhibited in cells expressing N17Rac1, N17Cdc42, L61Rac1, or L61Cdc42, but was not affected in cells expressing WT RhoA, N19RhoA, or L63RhoA (Figure 3G, 3H, 3I), these data was reinforced by results obtained using inhibitor drugs (Y27632, IPA-3) (Figure 3C). All of these results indicate that RHO-family-GTPases play an important role in TGEV entry. Specifically, Rac1 and Cdc42 are involved in the regulation of cofilin early in TGEV infection, while RhoA is not involved.

### The PI3K is critical for the entry of TGEV and is involved in cofilin phosphorylation early in infection

**Figure 4:**
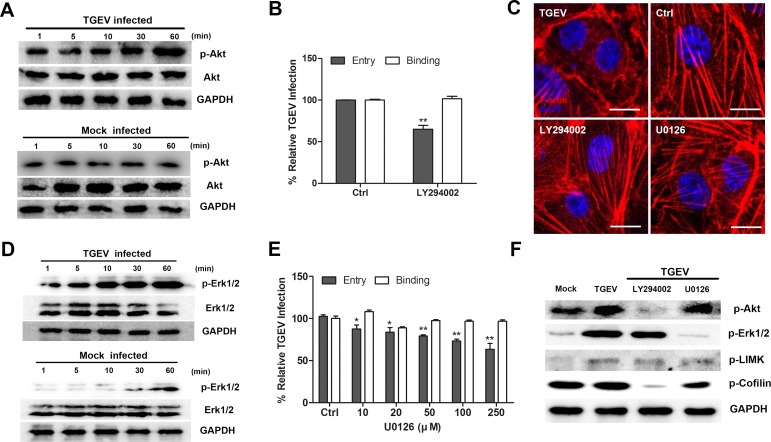
The PI3K-Akt pathway is involved in the regulation of actin cytoskeleton by cofilin **A.** Activation of Akt by TGEV entry. Cells lysates were immunoblotted with anti-p-Akt and anti-Akt mAbs. **B.** Activation of ERK1/2 during TGEV entry. Cells lysates were immunoblotted with anti-p-ERK1/2 and anti-ERK1/2 mAbs. **C.** PI3K inhibitor LY294002 inhibits TGEV entry. IPEC-J2 cells were pretreated with LY294002 (50 μM) at 37°C for 1 h. Binding and entry assays are described in materials and methods. **D.** MEK1/2 inhibitor U0126 inhibits TGEV entry. IPEC-J2 cells were pretreated with U0126 at different concentrations at 37°C for 1 h. **E.** LY294002 and U0126 inhibit the activation of downstream pathways. Cells were treated with LY294002 (50 μM) or U0126 (250 μM) at 37°C for 1 h, then infected by TGEV. 30 minutes after infection, cell lysates were analyzed for the phosphorylation of Akt, ERK1/2, LIMK, and Cofilin. **F.** LY294002 and U0126 protect the actin cytoskeleton. Cells were treated with LY294002 (50 μM) or U0126 (250 μM) at 37°C for 1 h, then infected by TGEV. After 1 h, cells were fixed and stained with phalloidin-TRITC and observed by confocal fluorescence microscopy. Statistical significance was assessed by Student's *t*-test (*). Differences were considered to be significant at 0.01 < *p* < 0.05, (**) *p* < 0.01. All experiments were performed independently three times. Scale bar = 20 μm. TGEV at a multiplicity of infection of 2 (MOI = 2).

Neither PI3K modulation of the Rho-GTPase family, nor the involvement of PI3K/Akt pathway early in coronavirus infection, have been demonstrated conclusively. To investigate the role of PI3K/Akt pathway in TGEV entry, we monitored the level of p-Akt in cells shortly after infection. From 10 mpi to 60 mpi, the level of p-Akt increased in TGEV infected cells (Figure 4A). In cells pretreated with LY294002, a highly specific inhibitor of PI3K, virus binding was unaffected, but TGEV entry was reduced 40% compared with untreated cells (Figure 4B). Examination by confocal fluorescence microscopy demonstrated that LY294002 inhibited the destruction of F-actin early in TGEV infection (Figure 4C).

We previously have showed that attenuated TGEV (STC3 strain) and PEDV (CV777 strain) could regulate the F-actin *via* the MAPK pathways [23]. In this study, we found that TGEV infection induced the activity of ERK1/2 at 5 mpi and increased it thereafter (Figure 4D). U0126, a specific inhibitor of MEK1/2, inhibited TGEV entry in a dose dependent manner but did not affect the ability of the virus to bind to cells (Figure 4E). U0126 also inhibited the destruction of F-actin by TGEV infection, as observed when cells were examined by confocal fluorescence microscopy (Figure 4C).

The phosphorylation of Akt, LIMK and cofilin were inhibited when cells were treated with PI3K inhibitor LY294002 (Figure 4F), suggesting that the PI3K/Akt pathway is involved in the regulation of actin cytoskeleton by cofilin. ERK1/2, LIMK and cofilin phosphorylation were inhibited when cells were treated with MEK1/2 inhibitor U0126, but the activation of ERK1/2 was not inhibited by LY294002. These results indicate that the MEK/ERK pathway also involve in the regulation of the actin cytoskeleton by cofilin, but it is not activated by PI3K/Akt pathway.

### EGFR is involved in cofilin phosphorylation early in infection

**Figure 5:**
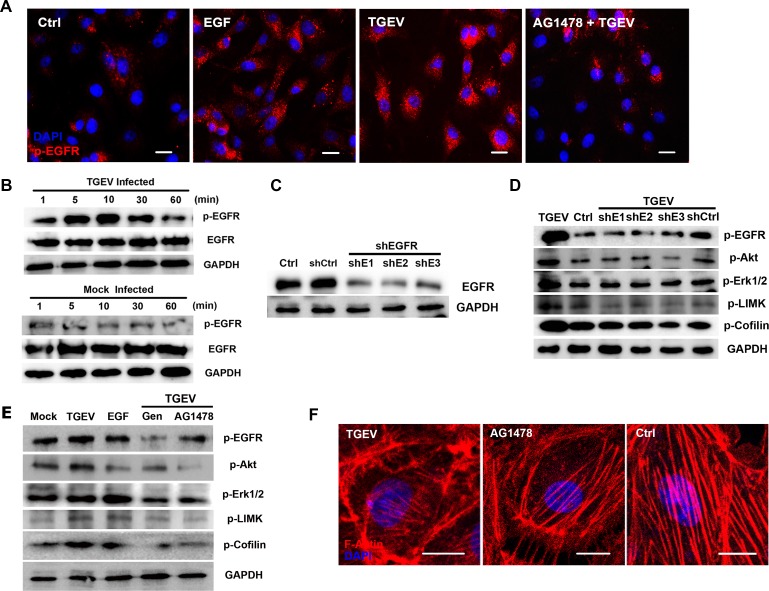
EGFR is involved in the regulation of actin cytoskeleton by cofilin **A.** TGEV infection induces the activation of EGFR and clustering. IPEC-J2 cells were incubated with TGEV at 4°C for 1 h. then shifted to 37°C. Cells were fixed 10 mpi, and stained with anti-p-EGFR antibody. Cells pretreated with EGF (100 ng ml^−1^) or AG1478 (100 nM) at 37°C were used as controls. **B.** Activation of EGFR by TGEV entry. Cells lysates were immunoblotted with anti-p-EGFR and anti-EGFR mAbs. **C.** EGFR shRNAs verification. The EGFR targeting shRNAs significantly inhibited EGFR expression, as verified by Western blotting. **D.** EGFR shRNAs inhibit the activation of downstream pathways. Cells were treated with EGFR shRNA-expressing lentiviral particles at 37°C for 48 h. After infection by TGEV for 30 min, cell lysates were analyzed for the phosphorylation of EGFR, Akt, ERK1/2, LIMK, and cofilin. **E.** RTK and EGFR inhibitors inhibit the activation of downstream pathways. Cells were treated with Gen (250 μM) or AG1478 (100 nM) at 37°C for 1 h. After infection by TGEV for 10 min, cell lysates were analyzed for the phosphorylation of Akt, ERK1/2, LIMK, and Cofilin. **F.** AG1478 protects the actin cytoskeleton. Cells were treated with AG1478 (100 nM) at 37°C for 1 h then infected with TGEV. After 1 h, cells were fixed and stained with phalloidin-TRITC and observed by confocal fluorescence microscopy. Statistical significance was assessed by Student's *t*-test. Differences were considered significant at (*) 0.01 < *p* < 0.05, (**) *p* < 0.01. All experiments were performed independently three times. Scale bar = 20 μm. TGEV at a multiplicity of infection of 2 (MOI = 2).

We have concluded that the PI3K/Akt pathway involve in TGEV infection, but the involvement of EGFR have not been demonstrated. To investigate the role of EGFR more closely, we stained cells with an anti-p-EGFR antibody after various treatments and then observed them using fluorescence microscopy. At 10 mpi, both TGEV and EGF increased the phosphorylation level of EGFR, but EGFR specific inhibitor AG1478 significantly impaired EGFR phosphorylation (Figure 5A). Western blotting results confirmed that the phosphorylation level of EGFR increased from 5 mpi to 30 mpi and reaching a peak at 10 mpi (Figure 5B).

To study the relationship between EGFR and downstream signaling pathways, we conducted experiments using different inhibitors and EGFR shRNAs. As expected, EGFR-targeting shRNAs significantly inhibited EGFR expression (Figure 5C). The activation of Akt, LIMK, and cofilin phosphorylation were inhibited by EGFR shRNAs (Figure 5D), as well as with RTK inhibitor Gen, and EGFR inhibitor AG1478 (Figure 5E). The activity of ERK1/2 was also inhibited by EGFR shRNAs, Gen, and AG1478. TGEV infection caused the dissolution of stress fibers and stimulated the formation of membrane protrusions, but these phenomena were blocked by AG1478 treatment (Figure 5F). Taken together, these results indicate that EGFR is involved in the entry of TGEV and the activation of downstream pathways early in the infection process.

### EGFR is involved early in TGEV infection

**Figure 6:**
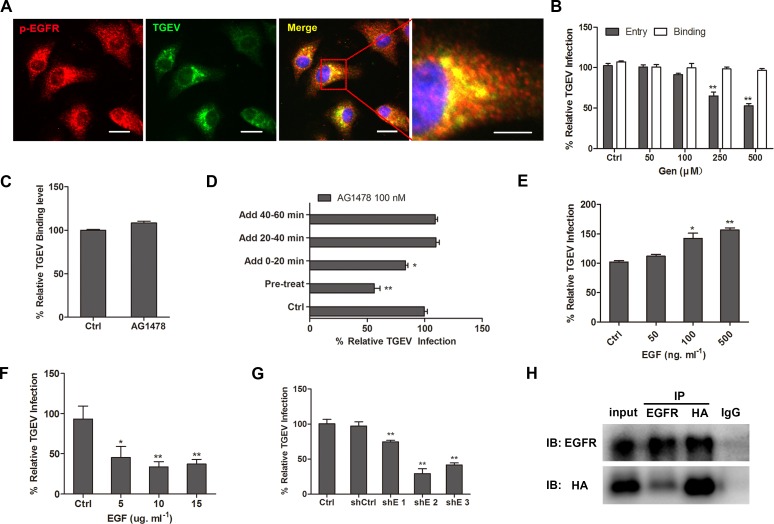
EGFR is involved in TGEV entry **A.** TGEV is co-localized with EGFR. After 10 mpi, cells were fixed and stained with mouse anti-TGEV mAb, followed by DyLight 488-conjugated goat anti-mouse IgG and anti-p-EGFR pAb, followed by Dylight 594-conjugated goat anti-rabbit IgG. Cells were examined by IF microscopy. **B.** The RTK specific inhibitor, genistein (Gen), inhibits TGEV entry in a dose dependent manner. Cells were pretreated with different concentrations of genistein. TGEV binding and entry assays are described in materials and methods. **C.** and **D.** The EGFR specific inhibitor, AG1478, inhibits TGEV entry. IPEC-J2 cells were pretreated with AG1478 for 1 h at 37°C. Alternatively, IPEC-J2 cells were incubated with TGEV at 4°C for 1 h, unbound viruses were removed, cells were shifted to 37°C, and AG1478 was added at 0-20 mpi, 20-40 mpi, and 40-60 mpi. **E.** The activation of EGFR promotes TGEV entry. Serum-starved IPEC-J2 cells were pretreated with different low concentrations of EGF at 37°C for 1 h, cells were infected with TGEV for 1 h and entry of TGEV was evaluated by real-time PCR. **F.** The internalized of EGFR inhibits TGEV entry. Serum-starved IPEC-J2 cells were pretreated with different high concentrations of EGF at 37°C for 1 h, cells were infected with TGEV for 1 h and entry of TGEV was evaluated by real-time PCR. **G.**EGFR shRNAs block TGEV entry. Cells were transfected with lentiviral particles expressing EGFR shRNAs. 48 h post-infection, cells were infected with TGEV for 1 h, and entry of TGEV was evaluated by real-time PCR. **H.** TGEV S1 interacts with EGFR. Cells were co-transfected with plasmids expressing TGEV S1-HA and EGFR. Immunoprecipitation and immunoblotting were performed to examine interactions between TGEV S1 and EGFR. Statistical significance was assessed by Student's *t*-test. Differences were considered significant at (*) 0.01 < *p* < 0.05, (**) *p* < 0.01. All experiments were performed independently three times. Scale bar = 20 μm. TGEV at a multiplicity of infection of 2 (MOI = 2).

Using fluorescence microscopy, we found that the spike protein of TGEV co-localized with p-EGFR (Figure 6A). The RTK specific inhibitor genistein (Gen) had no inhibition effect on the adhesion of TGEV, but inhibited TGEV entry in a dose dependent manner (Figure 6B). Although EGFR specific inhibitor AG1478 had no inhibition effect on the adhesion of TGEV (Figure 6C), when IPEC-J2 cells were pretreated with AG1478 for 1h at 37°C, TGEV entry was significantly decreased (Figure 6D). AG1478 appeared to act only at early stage of TGEV entry, shortly after the virus bound to the cell (Figure 6D). When serum-starved cells were pretreated with low concentration of EGF at 37°C for 1h, EGFR was activated and TGEV entry was increased significantly (Figure 6E). When serum-starved cells were pretreated with high concentration of EGF at 37°C for 1h, most of EGFR internalized into the cytoplasm and TGEV entry was decreased significantly (Figure 6F). EGFR targeting shRNAs significantly decreased TGEV entry (Figure 6G). The interaction between TGEV S1 and EGFR were confirmed in HEK293T cells that were co-transfected with plasmids expressing TGEV S1-HA and EGFR (Figure 6H), providing further support that TGEV S1 directly combined with EGFR. Most of EGFR occured in the cell membrane in normal cells, part of EGFR internalized into the cytoplasm upon low concentration EGF (100ng/ml) stimulation, most of EGFR internalized into the cytoplasm upon high concentration EGF(10ug/ml) stimulation, both TGEV particles and EGFR internalized into the cytoplasm upon TGEV infection (Figure S3). Taken together, these results indicate that EGFR acts as a membrane promoter for TGEV entry.

### Lipid rafts cluster in the cell membrane and activate downstream signaling pathways early in TGEV infection

**Figure 7:**
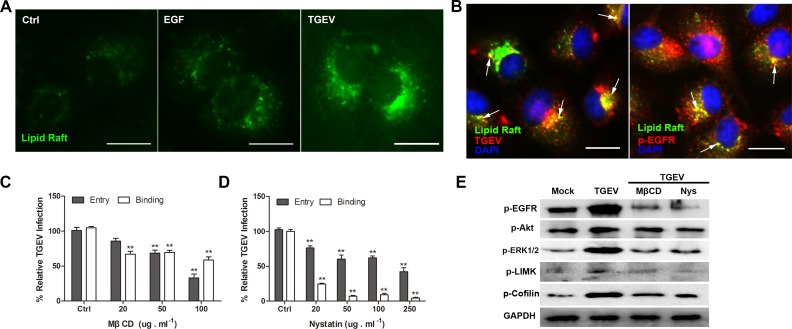
Lipid rafts are involved early in infection by TGEV **A.** Attachment of TGEV causes the clustering of plasma membrane lipid rafts. **B.** Both TGEV and EGFR are co-localized with GM1 at the cell surface. **C.** and **D.** Cholesterol-sequestering drugs inhibit TGEV entry. Cells were pretreated with different concentrations of MβCD or nystatin at 37°C for 1 h. **E.** Destruction of lipid rafts inhibits the activation of downstream signaling pathways. Differences were considered significant at (*) 0.01 < *p* < 0.05, (**) *p* < 0.01. All experiments were performed independently three times. Scale bar = 20 μm. TGEV at a multiplicity of infection of 2 (MOI = 2).

When EGFR in the lipid rafts is stimulated, the endocytosis of membrane microdomains *via* clathrin-dependent and clathrin-independent mechanisms occurs [24, 25]. Lipid rafts are mainly composed of sphingolipid and cholesterol [26]. Ganglioside GM1 serves as lipid rafts marker and can be labeled with fluorescein isothiocyanate (FITC)-conjugated cholera toxin beta subunit (CtxB) [18, 19]. Using this reagent, we found that lipid rafts distributed evenly in the cell membrane of untreated cells. In contrast, the lipid rafts clustered in EGF treated or TGEV infected cells (Figure 7A). These results show that TGEV infection causes lipid rafts function as a platform to concentrate APN and EGFR.

**Figure 8:**
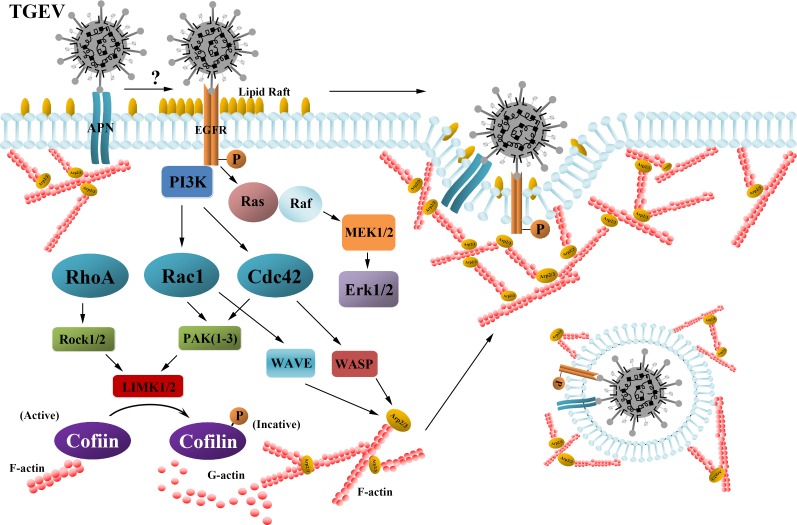
The signaling pathway of regulating the actin dynamics by cofilin and initiating TGEV entry Early after infection, TGEV stimulates the phosphorylation of cofilin and the polymerization of F-actin through the EGFR-PI3K-Rac1/Cdc42-PAK-LIMK signaling pathway. EGFR is another receptor for TGEV invasion, and is involved in the regulation of actin dynamics and TGEV invasion. Lipid rafts are clustered and act as signaling platforms for EGFR-mediated signaling.

To analyze whether TGEV or EGFR is present in lipid rafts upon TGEV infection, co-localization experiments were performed. Lipid rafts were labeled with FITC-conjugated CtxB, TGEV was stained with mouse anti-TGEV mAb, and EGFR was stained with rabbit anti-EGFR pAb. Both TGEV and EGFR co-localized with GM1 at the cell surface (Figure 7B). Therefore, we conclude that EGFR and the attaching TGEV particles are located together in the lipid rafts during infection.

For studying the role of lipid rafts in the entry process of TGEV, both methyl-β-cyclodextrin (MβCD) and nystatin were used to remove the cholesterol from the cell membrane. The effect of these inhibitors on cell viability were shown in S1 Fig. Cells were pretreated with different concentrations of MβCD or nystatin at 37°C for 1h prior to infection, both TGEV binding and entry levels were inhibited in a dose dependent manner compared with mock control cells (Figure 7C, 7D). These results suggest that lipid rafts play an important role in TGEV binding and entry.

During the early phase of TGEV infection, the destruction of lipid rafts inhibited the activation of EGFR, Akt, and LIMK, and also inhibited the phosphorylation of cofilin (Figure 7E). These results indicate that lipid rafts function as a platform to induce the activation of EGFR and the downstream PI3K/Akt signaling pathway, so the clustering of lipid rafts involve in the regulation of actin cytoskeleton by cofilin.

## DISCUSSION

The role of actin cytoskeleton in TGEV entry process is unclear. We had shown that TGEV induced F-actin rearrangement and caused membrane ruffles. TGEV entry required F-actin gathered at the cell membrane. We identified cofilin as a critical factor for regulating cytoskeleton dynamics and TGEV infection. Our results also indicate that TGEV binding induces the phosphorylation of cofilin through the EGFR-PI3K-Rac1/Cdc42-PAK-LIMK signaling pathways, resulting in actin polymerization around the cell membrane and foming multiple poutrusions.

The highly dynamic nature of actin cytoskeleton affects every stage of the viral life cycle, from entry through assembly to release. Virion attachment to cells can stimulate the extension of cell surface protrusions [4]. Human Immunodeficiency Virus (HIV) and Herpes simplex virus (HSV) virions cause cell surface extensions and viral-entry receptor clustering [27, 28]. The entry of vesicular stomatitis virus (VSV) virions is enhanced by the formation of actin filaments, and actin filaments are recruited to increase the size of the endocytic structure [29]. Early in the infection of TGEV, we observed the rearrangement of cortical actin and the formation of ruffles and protrusions in the cell membrane, as well as EGFR and lipid rafts clustering in the cell membrane, the dissolution of actin stress fibers and their relocalization around the cell plasma membrane. These changes appear to mediate TGEV entry.

Cofilin has a central role in controlling actin dynamics by regulating actin polymerization and depolymerization through its severing activity, as well as by inducing dendritic nucleation [6]. The regulation of cofilin activity is a component of pathogen-mediated actin remodeling [30, 31]. We conclude that phosphorylation of cofilin is critical for TGEV entry. Cofilin can be found in multiple cellular compartments, including the cytoplasm and nucleoplasm [32, 33]. A nuclear localization signal (amino acid residues 30-34) with high homology to the consensus nuclear localization signal sequence of SV40 large T-antigen is found in the cofilin sequence [34]. The nuclear translocation of cofilin is regulated by reversible phosphorylation of Ser-3 [35]. The phosphorylation of cofilin inhibits its F-actin severing activity. We found that p-cofilin was distributed mainly in the nucleus of normal IPEC-J2 cells, however, early after TGEV infection, most p-cofilin was found in the plasma membrane, derived both from the phosphorylation of cofilin and the migration of p-cofilin from the nucleus. Late in infection, a portion of the p-cofilin was dephosphorylated, while the remaining p-cofilin returned to the nucleus. Cofilin supports ARP2/3 complex-mediated actin branching by creating new actin filaments through its F-actin severing activity, making the actin filaments more stable at the cell membrane [36]. Cofilin is required for the early polymerization response to growth factor stimulation and the formation of protrusions, although the ARP2/3 complex is not involved in this process [37, 38]. The inhibition of ARP2/3 caused the actin filaments to bind together more loosely, resulting in increased entry of TGEV. We conclude that the structure of the cortical actin filaments, which underline the cell membrane, plays an important role in TGEV early infection. The regulation of cofilin activity acts as a molecular switch to provide a functional link between actin cytoskeleton rearrangement and the TGEV entry process.

The actin cytoskeleton is mainly regulated by RHO-family-GTPases, which control the signaling pathway that links membrane receptors to the cytoskeleton [4]. The RHO family of GTPases contain more than twenty members. Rac1, a primary example, induces membrane ruffles or lamellipodia [39]. PI3K/Akt is the main activation pathway for Rac1 in many cell types [40]. Cofilin is inactivated by Rac1 and Cdc42 through PAK and LIMK. The entry of Ebola virions and VSV pseudovirions are decreased when RhoA is overexpressed [41]. RhoA activation by PI3K leads to the formation of stress fibers [4]. Constitutively activated mutant L63RhoA caused the formation of stress fibers, and prevented the destruction of stress fibers caused by TGEV and reduced the amount of F-actin gathered around the cell membrane. The inhibition of ROCK had no effect on the entry of TGEV or the phosphorylation of cofilin. Both constitutively inactivated mutant N17Rac1 and constitutively activated mutant L61Rac1 impeded the formation of lamellipodia and membrane ruffles, and both constitutively inactivated mutant N17Cdc42 and constitutively activated mutant L61Cdc42 impeded the formation of protrusive filopodia. These effects inhibited TGEV entry.

The activation of the PI3K signaling pathway is involved in the early steps of virus infection, such as receptor/co-receptor engagement and virus internalization [42]. PI3K is a key mediator of the signaling pathway that regulates actin cytoskeleton reorganization and polarized cell migration [7]. This study shows that the PI3K/Akt pathway is involved in the cellular entry of TGEV, and is activated by EGFR. Our previous studies indicate that MAPK pathways are activated early in TGEV infection (for example, the Ras-mediated activation of the Raf/MEK/ERK cascade). MAPK pathways can also be activated by the EGFR-PI3K-RhoGTPases signaling pathway [43, 44]. These data demonstrate that TGEV also activates MAPK pathways to regulate the actin cytoskeleton through EGFR early in infection.

EGFR, a member of the ErbB receptor family, is expressed in many cell types. The primary signaling pathways activated by RTKs include PI3K/Akt, Ras/Raf/ERK1/2, and signal transduction and activator of transcription (STAT) pathways. Our data provides evidence that EGFR acts as a signaling-promoter that is involved in the regulation of actin and TGEV internalization. Human papillomavirus (HPV) infection induces filopodia formation and the dissolution of stress fibers by 10 mpi, and HPV entry is also blocked by RTK and PI3K inhibitors [45]. We find that the EGFR specific inhibitor AG1478 and PI3K specific inhibitor Y27632 block stress fiber dissolution, suggesting that the EGFR and PI3K are involved in the signaling pathway that regulates the assembly of F-actin. Our results indicate that EGFR is activated by TGEV binding and promotes TGEV uptake into host cells. It has been reported that a 200-kDa protein in ST cells and in villous enterocytes in newborn pigs may be a second receptor for TGEV and contribute to the age sensitivity of these animals to the virus [46], but the identity of this 200-kDa receptor has not been determined. Although EGFR is a 170-kDa transmembrane protein, we have found that it interacted with TGEV S1 protein, and therefore should be considered as a factor in determining the high level of susceptibility of newborn pigs to TGEV.

The clustering of large lipid rafts is caused by protein modifications such as phosphorylation, which increase the number of protein-protein interactions [47]. We found that TGEV stimulated the clustering of large lipid rafts, and that activated EGFR, and TGEV virions were associated with the rafts. The clustering of the lipid rafts functions as a signaling platform, but the activation of downstream signaling pathways were inhibited by agents that remove the rafts. The integrity of lipid rafts is essential for the activation of Src kinase and the PI3K/Akt pathway [48]. Based on these data and our results, we hypothesize that TGEV induces the clustering of lipid rafts by the binding of spike protein to APN and EGFR. The activated EGFR then transfers the signal to effector proteins downstream.

APN is the specific receptor for TGEV. Our experiments identified EGFR as another promoter for TGEV entry. It is not known whether APN and EGFR are related in any way, or whether other TGEV receptors exist. Actin plays an important role in the endocytosis and vesicle transport [49], and the cytoskeleton is involved in different stages of both clathrin- and caveola-mediated endocytosis. Understanding the main endocytic pathway of TGEV, and the molecular mechanisms regulating this process, will require further investigation. These findings are conducive to enhancing our understanding of the entry mechanism of TGEV and providing a potential target for the development of new anti-TGEV therapies.

## MATERIALS AND METHODS

### Cell lines

IPEC-J2 cells are porcine intestinal columnar epithelial cells that were isolated from the middle jejunum of neonatal piglets. IPEC-J2 cells were purchased from DSMZ (Germany). HEK293T cells were purchased from ATCC (United States). Both IPEC-J2 and HEK293T cells were maintained in Dulbecco's Modified Eagle's Medium (DMEM) with high glucose, HEPES supplemented with 10% fetal bovine serum (FBS, GIBCO), 1% penicillin-streptomycin (Invitrogen) at 37°C in a 5% CO_2_ incubator (Thermo Scientific).

### Virus and infection

Transmissible gastroenteritis virus (strain SHXB) was isolated in Shanghai, China. The complete genome sequence for TGEV SHXB is available in GenBank (KP202848.1) [50]. For the binding assays, cells were incubated with TGEV at a multiplicity of infection of 2 (MOI = 2) for 1 h at 4°C, and washed with PBS (pH7.2 at 4°C) three times to remove unbound virus, then we added the Trizol to collect the samples. For the entry assays, cells were incubated with TGEV at MOI = 2 for 1 h at 4°C and washed with PBS (pH7.2 at 4°C) three times to remove unbound virus, then maintained in maintenance medium (DMEM supplemented with 2% FBS and 1% penicillin-streptomycin) at 37°C in a 5% CO_2_ incubator, after the indicated time, cells were washed with acidic PBS (pH 3.0 at 4°C) to remove the virus bound to the cell membrane (not enter the cell), then we added the Trizol to collect the samples.

### RNA extraction and RT-PCR

Total RNA from IPEC-J2 cells infected with TGEV was extracted using TRIzol Reagent (Invitrogen) according to the manufacturer's instructions. The cDNA was generated by reverse transcription using HiScript^TM^ QRT SuperMix for qPCR (Vazyme) according to the manufacturer's instructions. TGEV entry and binding were assessed by detecting the viral nucleoprotein (N) gene using quantitative RT-PCR with the TaKaRa SYBR Green qPCR Kit (TaKaRa). Primer sequences were as follows: N-F (sense), 5′-CAATTCCCGTGGTCGGAAGA-3′; N-R (antisense), 5′-TTTACGTTGGCCCTTCACCA-3′; GAPDH-F, 5′-TCATCATCTCTGCCCCTTCT-3′; GAPDH-R, 5′-GTCATGAGTCCCTCCACGAT-3′. PCR products were purified using a Gel Extraction Kit (Omega), and cloned into the pJET1.2 vector (Thermo). Plasmids were diluted serially and used as standards for quantitative analysis. The initial copy number of TGEV N gene and GAPDH in each group was calculated using the following formula: X0 = −K log Ct + b, where X0 is the initial copy number, K, Ct, and b refer to the slope rate, cycle threshold, and constant, respectively. Quantitative real-time PCR was performed with the ABI PRISM 7500 Detection System (Applied Biosystems, Foster, USA).

### Western blotting

At indicated times post infection, cells were washed with PBS and lysed in radioimmunoprecipitation assay (RIPA) buffer (Thermo Scientific). Phosphatase inhibitor and Protease inhibitor (Thermo Scientific) were added in the RIPA according to the manufacturer's instructions. The concentration of the lysates were determined by a Pierce BCA Protein Assay kit based on the bicinchoninic acid spectrophotometric method (Thermo Scientific). After centrifugation at 12000×g for 10 min, the supernatant (15-50 ug of protein) was fractionated by SDS-PAGE (10%-12% gradient), the separated proteins were transferred to PVDF (Merck Millipore), the membranes were blocked for 2h in Tris-buffered saline (TBS) containing 5% nonfat dry milk, and reacted with the indicated primary antibodies at 4°C overnight. Membranes were exposed to species specific horseradish peroxidase (HRP)-conjugated secondary antibodies (dilution 1:5000) followed by enhanced chemiluminescence (ECL, Thermo Scientific) detection by autoradiography. Western blotting was quantified by Quantity One (Quantity One 1-D Analysis Software 170-9600, Bio-Rad). The intensity of the bands in terms of density was measured and normalized against GAPDH expression. All data were expressed as means ± SD of three independent experiments.

### Plasmids construction

To construct the pcDNA3.1 vectors, RhoA, Rac1 and Cdc42 sequences were inserted into the Nhe I/HindIII site. pLVX-DsRed-Monomer-N1 is an HIV-1-based, lentiviral expression vector that express the gene of interest fused to DsRed-Monomer (Clontech). RhoA, Rac1 and Cdc42 sequences were inserted into the EcoRI/BamHI site. To construct vectors expressing RhoA, Rac1, and Cdc42 mutants (constitutively-activated mutants pLVX-L63RhoA, pLVX-L61Rac1, and pLVX-L61Cdc42; constitutively-inactivated mutants pLVX-N19RhoA, pLVX-N17Rac1, and pLVX-N17Cdc42), sequences were amplified from pcDNA3.1-RhoA, Rac1, and Cdc42 respectively. The constitutively-phosphorylated mutant (inactivated) pLVX-CofilinS3E, and the constitutively non-phosphorylated mutant (activated) pLVX-CofilinS3A, were amplified from pcDNA3.1-cofilin. All mutants were generated using the Mut Express II Fast Mutagenesis Kit (Vazyme) according to the manufacturer's instructions. All constructs were verified by DNA sequencing. The primers used in PCR and for the site-directed mutagenesis are described in Supplemental Tables S3 and S4.

### Lentivirus-mediated RNA interference depletion experiments

pLVX-shRNA2 is an HIV-1-based, lentiviral expression vector designed to express a small hairpin RNA (shRNA) for RNA interference (RNAi) studies (Clontech). The best silencing efficiency was observed with clone NM_001004043 (porcine cofilin) and NM_214007 (porcine EGFR). Targeting sequences are described in Supplemental Table S5. HEK293T cells were transfected with 1 μg of specific expression plasmid per 10^6^ cells using the X-tremeGENE HP DNA Transfection Reagent (Roche) according to the manufacturer's instructions, with Opti-MEM (Invitrogen) included in T-25 cell culture flask. Lentivial particles (MOI = 1) were added to the IPEC-J2 cells, and gently mixed.

### Transmission electron microscopy

IPEC-J2 cells were cultivated on T-25 cell culture flask and incubated with TGEV at MOI = 2 for 1h at 4°C, then shifted to 37°C for 30 min. Samples were fixed in a 2.5% glutaraldehyde solution in PBS for 24h. After fixation, the monolayer was gently removed from the flask with a rubber policeman and the cell suspension was pelleted by low speed centrifugation (2500×g for 5min). The cells were then washed twice by centrifugation in 0.1 M PBS. The pellet of cells was then fixed overnight at 4°C and then prepared for transmission electron microscope (TEM) as described [51]. Samples were visualized using a Hitachi-7650 transmission electron microscope (TEM, Japan) at 120 kV.

### Immunofluorescence staining and microscopy

IPEC-J2 cells were grown on coverslips in 24-well tissue culture plates and incubated with TGEV at MOI=2 for 1 h at 4°C, then shifted to 37°C. At the indicated time points, cells were fixed in 4% paraformaldehyde for 15 min then permeabilised with 0.1% Triton X-100 for 5 min. Samples were blocked in 5% bovine serum albumin (BSA) for 20 min and incubated with primary antibodies (1:1000 for the Cell Signaling Technology antibody and Santa Cruz antibody) at 4°C overnight then incubated with fluorochrome-conjugated secondary antibodies (1:500) at room temperature for 1 h. To stain F-actin, phalloidin-TRITC (Green, Red) (Life) was added for 20 min at room temperature. Nuclei were stained using 1 ug/ml DAPI (4′,6′diamidino-2-phenylindole dihydrochloride)-PBS for 5 min at room temperature. Images were captured with a Zeiss LSM710 confocal laser-scanning microscopy system and a 40x objective lens. Top view images were prepared as compacted Z-Stack images of non-permeabilised cells, using ZEN 2012 (Zeiss) software. The co-localization of two channels was detected using the co-localization finder plug-in. X-y plane and z-axis views of confocal images were prepared using ZEN 2012 LE software from Zeiss, Germany.

For viral labeling, viruses were filtered with 0.22μm filter, and then clarified by centrifugation at 10,000×g for 2.5 h, followed by ultra-centrifugation using 20%, 40%, 60% sucrose gradient at 10,000×g for 2.5 h, viruses were labeled with the fluorescent probe DyLight 594 NHS Ester (Thermo Scientific) according to the manufacturer's instruction. Unincorporated dye was removed by using commercial fluorescent dye removal columns (Thermo Scientific).

To observe the clustering of lipid rafts in the cell membrane, IPEC-J2 cells were incubated with TGEV (MOI = 2) or EGF (100 ng ml^−1^), or left untreated at 4°C for 1 h. Subsequently, cells were incubated with FITC-conjugated cholera toxin beta subunit (CtxB) (30 ug ml^−1^) at 4°C for 1 h and then incubated at 37°C for 10 min. Cells were fixed and observed by IF microscopy. To observe the co-patching of lipid rafts with proteins of interest and for co-localization experiments, IPEC-J2 cells were incubated with TGEV (MOI = 2) at 4°C for 1 h, and then incubated with FITC-CtxB at 4°C for 1 h and at 37°C for 10 min. EGFR and TGEV were visualized by the common immunofluorescence staining protocol. The cells were fixed and stained with mouse anti-TGEV mAb followed by DyLight 594-conjugated goat anti-mouse IgG or rabbit anti-p-EGFR mAb followed by DyLight 594-conjugated goat anti-rabbit IgG. Cells were examined by IF microscopy.

### Immunoprecipitation

Cells were lysed in NP-40 lysis buffer (Beyotime). Protease inhibitor (Thermo Scientific) was added according to the manufacturer's instructions. After centrifugation at 12000×g for 10 min, supernatant was pretreated with protein G PLUS-Agarose (Santa Cruz) and normal IgG (from the same species as that of the immunoprecipitating antibody) at 4°C for 1 h to eliminate nonspecific binding to the agarose beads or IgG. The supernatant was incubated with immunoprecipitation antibody (IP) at 4°C for 8 h and incubated with fresh agarose beads at 4°C for another 3 h. The agarose beads were washed five times with PBS, and the immunoprecipitated proteins were analyzed by Western blotting with specific primary and secondary antibodies.

### Statistical analysis

All results are presented as means ± standard deviation from three independent experiments. Significant differences between control and experimental groups were analyzed using Student's *t* test. Differences were considered significant at * 0.01 < *p* < 0.05, ** *p* < 0.01.

## SUPPLEMENTARY MATERIAL FIGURES AND TABLES


