# Supplementary material for: The epidermal growth factor receptor regulates cofilin activity and promotes transmissible gastroenteritis virus entry into intestinal epithelial cells
Source: Oncotarget. 2016 Feb 25;7(11):12206–21. doi: 10.18632/oncotarget.7723 (PMC4914279; doi:10.18632/oncotarget.7723)
Supplement: Supplementary file 1 [file oncotarget-07-12206-s001.pdf]

The epidermal growth factor receptor regulates cofilin activity and promotes transmissible gastroenteritis virus entry into intestinal epithelial cells

Supplementary Material

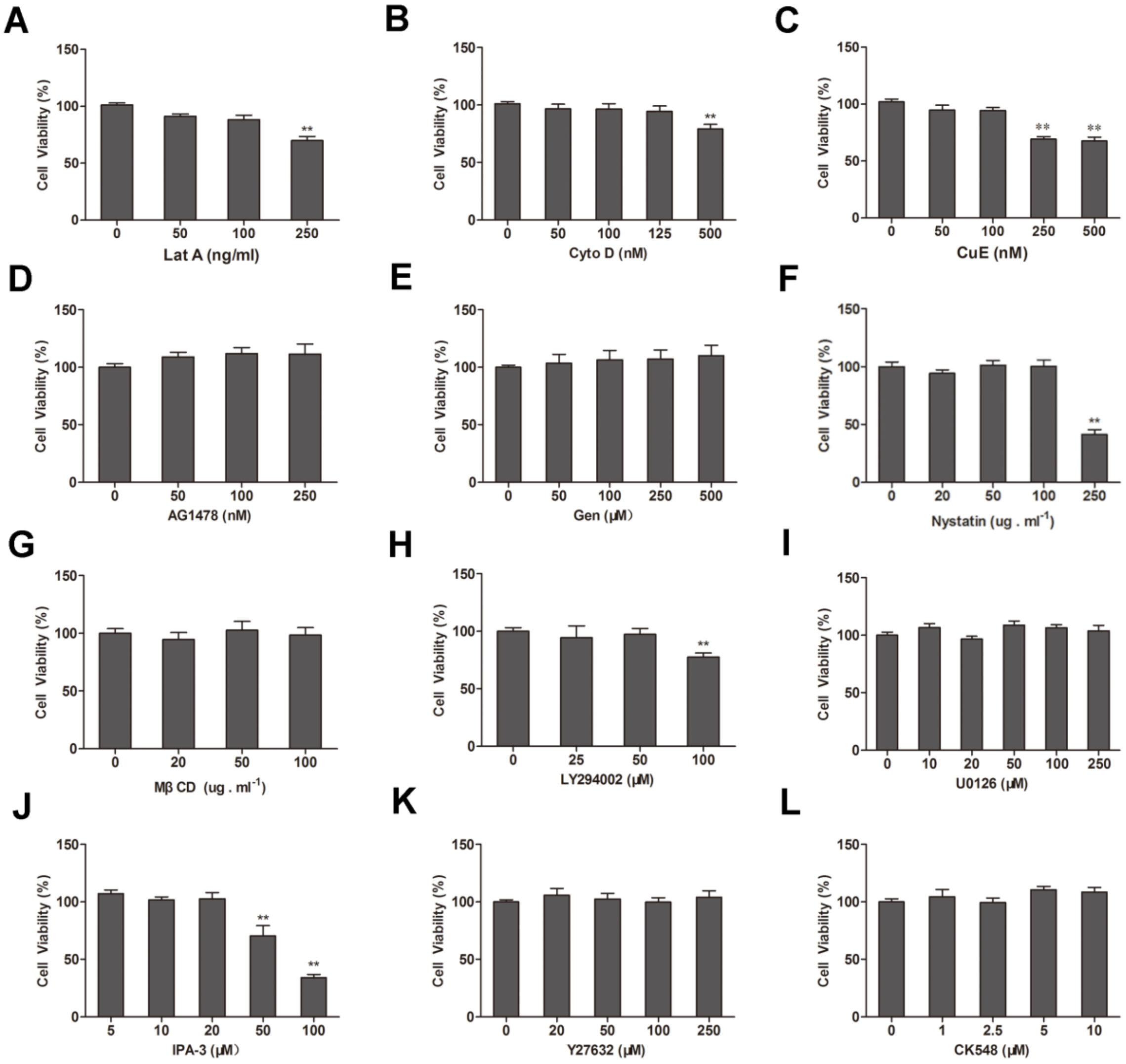

Figure S1: Cytotoxicity of chemical inhibitors. The cytotoxicity of chemical inhibitors were determined with a MTT [3-(4,5-dimethyl-2-thiazolyl)-2,5-diph enyl-2H-tetrazolium bromide] assay. Statistical significance was assessed by student's t-test (*) $0.01 < p < 0.05$, (**) $p < 0.01$, all of the experiments were performed for three separate experiments.

A

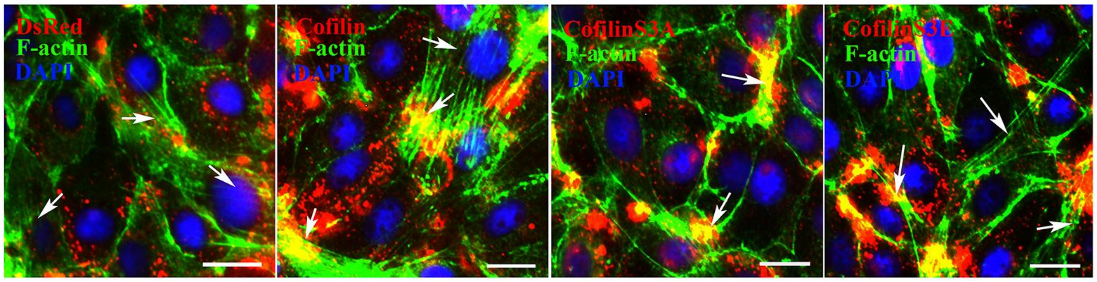

Figure S2: WT cofilin and mutants cofilins (S3A and S3E) destroy the arrangement of actin. Cells were transfected with lentiviral constructs expressing WT cofilin and mutant cofilins S3A and S3E. After incubation for 48 h, actin was stained with phalloidin-TRITC (Green).

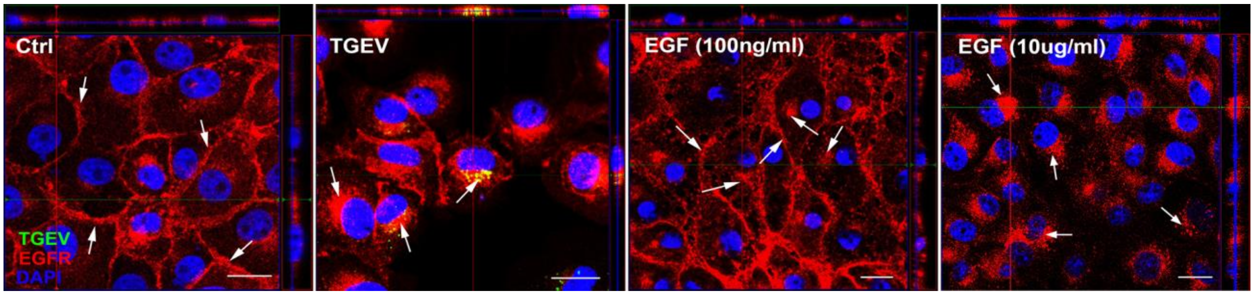

Figure S3: TGEV infection causes EGFR endocytosis. IPEC-J2 cells were infected with TGEV (MOI=2) or were stimulated with EGF (100ng/ml) at 4 °C for 1h then 37 °C for 30 min. The cells were fixed and stained with mouse anti-TGEV mAb followed by DyLight 488-conjugated goat anti-mouse IgG and rabbit anti-EGFR pAb followed by DyLight 594-conjugated goat anti-rabbit IgG, cells were examined by confocal microscope.

Table S1: List of pharmacological inhibitors.

Inhibitor	Specificity Functions	Supplier
**Latrunculin A**	sequesters actin monomers	Life
**Cytochalasin D**	actin depolymerizing agent	Life
**Cucurbitacin E**	stabilizes actin polymers	Sigma
**AG1478**	EGFR specific inhibitor	Sigma
**Genistein**	Receptor tyrosine kinase inhibitor	Sigma
**Nystatin**	sequesters and blocks cholesterol	Sigma
**MβCD**	Deplete cholesterol	Sigma
**LY294002**	Specific PI3K inhibitor	Sigma
**U0126**	MEK1/2 inhibitor	Sigma
**IPA-3**	PAK-1 inhibitor	Sigma
**Y27632**	ROCK-1 inhibitor	Sigma
**CK548**	Arp2/3 complex inhibitor	Sigma

Table S2: List of antibodies.

Name	Antibody information	Supplier	Catalog Number
**EGFR**	EGF Receptor (D38B1) XP® Rabbit mAb	CST	4267
**p-EGFR**	Phospho-EGF Receptor (Tyr1068) (D7A5) XP ® Rabbit mAb	CST	3777
**p-LIMK1**	Phospho-LIMK1 (Thr508) Rabbit mAb	CST	3841
**Akt**	Akt1 (C73H10) Rabbit mAb	CST	2938
**p-Akt**	Phospho-Akt (Ser473) (193H12) Rabbit mAb	CST	4058
**ERK1/2**	p44/42 MAPK (Erk1/2) (137F5) Rabbit mAb	CST	4695
**p-ERK1/2**	Phospho-p44/42 MAPK (Thr202/Tyr204) (D13.14.4E) XP™ Rabbit mAb	CST	4370
**Cofilin**	Cofilin Rabbit pAb	CST	3312
**p-Cofilin**	Phospho-Cofilin (Ser3) (77G2) Rabbit mAb	CST	3313
**HA**	Mouse monoclonal Antibody to HA	CMCTAG	AT0024
**TGEV**	TGE Virus Mouse mAb	Santa Cruz	sc-52436
**Dylight488**	Goat anti-Mouse IgG (H+L) Secondary Antibody, DyLight 488 conjugate	Thermo	35502
	Goat anti-Mouse IgG(H+L) Secondary Antibody, DyLight 594 conjugate	Thermo	35510
**Dylight594**	Goat anti-Rabbit IgG (H+L) Secondary Antibody, DyLight 594 conjugate	Thermo	35560

TableS3: List of primer sequences used in PCR

Name	Primer sequence(5'-3')	Vector
**Cofilin**	F: ACCCAAGCTGgctagcATGGCCTCCGGTGTG (NheI)<br>R: GCTCGGTACCaagcttCAAAGGCTTGCCCTCCAG (HindIII)	pcDNA3.1
**Cofilin**	F: CGAGCTCAAGCTTCgaattcATGGCCTCCGGTGTGGCT (EcoRI)<br>R: GTGGCGACCGGTggatccCAAAGGCTTGCCCTCCAG (BamHI)	pLVX-DsRed
**CofilinS3A**	F: CGAGCTCAAGCTTCgaattcATGGCCGCAGGTGTGGCT (EcoRI)<br>R: GTGGCGACCGGTggatccCAAAGGCTTGCCCTCCAG (BamHI)	pLVX-DsRed
**CofilinS3E**	F: CGAGCTCAAGCTTCgaattcATGGCCGAAGGTGTGGCT (EcoRI)<br>R: GTGGCGACCGGTggatccCAAAGGCTTGCCCTCCAG (BamHI)	pLVX-DsRed
**RhoA**	F: ACCCAAGCTGgctagcATGGCTGCCATCAGGAAG (NheI)<br>R: GCTCGGTACCaagcttCAAGACAAGGCACCCAGAT (HindIII)	pcDNA3.1
**RhoA**	F: CGAGCTCAAGCTTCgaattcATGGCTGCCATCAGGAAG (EcoRI)<br>R: GTGGCGACCGGTggatccCAAGACAAGGCACCCAGAT (BamHI)	pLVX-DsRed
**N19RhoA**	F: CGAGCTGAAGCTTCgaattcATGGCTGCCATCAGGAAG (EcoRI)<br>R: GCTCGGTACCaagcttCAAGACAAGGCACCCAGAT (HindIII)	pLVX-DsRed
**L63RhoA**	F: CGAGCTCAAGCTTCgaattcATGGCTGCCATCAGGAAG (EcoRI)<br>R: GTGGCGACCGGTggatccCAAGACAAGGCACCCAGAT (BamHI)	pLVX-DsRed
**Rac1**	F: ACCCAAGCTGgctagcATGCAGGCCATCAAGTGTGT (NheI)<br>R: GCTCGGTACCaagcttCAACAGCAGGCACTTCCTCT (HindIII)	pcDNA3.1
**Rac1**	F: CGAGCTCAAGCTTCgaattcATGCAGGCCATCAAGTGT (EcoRI)<br>R: GTGGCGACCGGTggatccCAACAGCAGGCACTTCCT (BamHI)	pLVX-DsRed
**N17Rac1**	F: CGAGCTGAAGCTTCgaattcATGCAGGCCATCAAGTGT (EcoRI)<br>R: GTGGCGACCGGTggatccCAACAGCAGGCACTTCCT (BamHI)	pLVX-DsRed
**L61Rac1**	F: CGAGCTCAAGCTTCgaattcATGCAGGCCATCAAGTGT (EcoRI)<br>R: GTGGCGACCGGTggatccCAACAGCAGGCACTTCCT (BamHI)	pLVX-DsRed
**Cdc42**	F: ACCCAAGCTGgctagcATGCAGACGATTAAGTGTGTTG (NheI)<br>R: GCTCGGTACCaagcttAATATACAGCACTTCCTTTTG (HindIII)	pcDNA3.1
**Cdc42**	F: CGAGCTCAAGCTTCgaattcATGCAGACGATTAAGTGTGTTG (EcoRI)<br>R: GTGGCGACCGGTggatccAATATACAGCACTTCCTTTTG (BamHI)	pLVX-DsRed
**N17Cdc42**	F: CGAGCTCAAGCTTCgaattcATGCAGACGATTAAGTGTGTTG (NheI)<br>R: GTGGCGACCGGTggattcAATATACAGCACTTCCTTTTG (BamHI)	pLVX-DsRed
**L61Cdc42**	F: CGAGCTCAAGCTTCgaattcATGCAGACGATTAAGTGTGTTG (EcoRI)<br>R: GTGGCGACCGGTggatccAATATACAGCACTTCCTTTTG (BamHI)	pLVX-DsRed
**TGEV-S1**	F: TCTAGCCCGGGCggatccTGTGCTAGTTATGTGGCT (BamHI)<br>R: ATCGTATGGGTAtctagaATTTGTATAATTATATATAGAG (XbaI)	pLVX-HA
**EGFR**	F: CTCAAGCTTCgaattcATGCGACGCTCCTGGGCG (EcoRI)<br>R: GTGGCGACCGGTggatccTCATGCCCCAGTAAGG (BamHI)	pLVX-DsRed

Table S4: List of primer sequences used in the site-directed mutagenesis

Name	Primer sequence(5'-3')
**CofilinS3A**	F: TAGCATGGCCGCAGGTGTGGCTGTCTCTGACGGGGTCATCAAAG<br>R:CAGCCACACCTGCGGCCATGCTAGCCAGCTTGGGTCTCCCTAT
**CofilinS3E**	F: TAGCATGGCCGAAGGTGTGGCTGTCTCTGACGGGGTCATCAAAG<br>R: CAGCCACACCTTCGGCCATGCTAGCCAGCTTGGGTCTCCCTAT
**N19RhoA**	F:TGTGGCAAGAATTGTTTGCTCATTGTCTTTAGCAAGGACCAAT<br>R:GAGCAAACAATTCTTGCCACAGGCTCCATCACCAACAATCAC
**L63RhoA**	F: ACAGCAGGACTGGAAGATTATGATCGTTTGAGGCCCCT<br>R: ATAATCTTCCAGTCCTGCTGTGTCCCACAAAGCCAACTCTA
**N17Rac1**	F:GTGGGTAAAAATTGCCTGATCAGTTACACGACCAATGC<br>R: CAGGAGGCAATTTTTACCCACAGCTCCGTCTCCCACCACCA
**L61Rac1**	F: ACGGCTGGACTAGAAGATTACGACCGGTTACGTCCCCTGTC<br>R: GTAATCTTCTAGTCCAGCCGTATCCCATAAGCCCAGATTCA
**N17Cdc42**	F: GTTGGTAAAAACTGTCTCCTGATATCCTACACGACAAACAAAT<br>R: TCAGGAGACAGTTTTTACCAACGGCACCATCGCCCACAACAAC
**L61Cdc42**	F: ACTGCAGGGCTAGAGGATTATGACAGATTACGACCGCTGAG<br>R: ATAATCCTCTAGCCCTGCAGTATCAAAAAGTCCAAGAGTAT

TableS 5: List of RNA interferences.

Name	mRNA accession	Targeting sequence
**Cofilin1**	NM_001004043	**shCof1:**GGGTCATCAAAGTGTTCAATG<br>**shCof2:** GGTCATCAAAGTGTTCAATGA<br>**shCof3:** GCGAGGACAAGAAGAACATCA<br>**shCof ctrl:** CAAAGTGTTCAATGAGGTCAT
**EGFR**	NM_214007.1	**shEGFR1:** GCAGATCATCCGAGGAAATG<br>**shEGFR2:** GCCATGGGCCACATCTGTAAC<br>**shEGFR3:** GGGCATGAACTATCTGGAAGA<br>**shEGFR ctrl:** GCCACATCTGTAACGCCATGG
